# Provider-Initiated HIV Testing and Counselling in Rwanda: Acceptability among Clinic Attendees and Workers, Reasons for Testing and Predictors of Testing

**DOI:** 10.1371/journal.pone.0095459

**Published:** 2014-04-17

**Authors:** Felix R. Kayigamba, Mirjam I. Bakker, Judith Lammers, Veronicah Mugisha, Emmanuel Bagiruwigize, Anita Asiimwe, Maarten F. Schim. van der Loeff

**Affiliations:** 1 INTERACT, Kigali, Rwanda; 2 Royal Tropical Institute, KIT Biomedical Research, Amsterdam, The Netherlands; 3 Academic Medical Center (AMC), Amsterdam, The Netherlands; 4 ICAP, Mailman School of Public Health, Columbia University, Kigali, Rwanda; 5 Ruhengeri hospital, Ministry of Health, Kigali, Rwanda; 6 Ministry of Health, Kigali, Rwanda; 7 Amsterdam Institute of Global Health and Development (AIGHD), Academic Medical Center (AMC), Amsterdam, The Netherlands; 8 Center for Infection and Immunity Amsterdam (CINIMA), AMC, Amsterdam, The Netherlands; 9 Public Health Service of Amsterdam (GGD), Amsterdam, The Netherlands; International AIDS Vaccine Initiative, United States of America

## Abstract

**Introduction:**

Routine provider-initiated HIV testing and counselling (PITC) may increase HIV testing rates, but whether PITC is acceptable to health facility (HF) attendees is unclear. In the course of a PITC intervention study in Rwanda, we assessed the acceptability of PITC, reasons for being or not being tested and factors associated with HIV testing.

**Methods:**

Attendees were systematically interviewed in March 2009 as they left the HF, regarding knowledge and acceptability of PITC, history of testing and reasons for being tested or not. Subsequently, PITC was introduced in 6 of the 8 HFs and a second round of interviews was conducted. Independent factors associated with testing were analysed using logistic regression. Randomly selected health care workers (HCWs) were also interviewed.

**Results:**

1772 attendees were interviewed. Over 95% agreed with the PITC policy, both prior to and after implementation of PITC policy. The most common reasons for testing were the desire to know one’s HIV status and having been offered an HIV test by an HCW. The most frequent reasons for not being tested were known HIV status and test not being offered. In multivariable analysis, PITC, age ≥15 years, and not having been previously tested were factors significantly associated with testing. Although workload was increased by PITC, HIV testing rates increased and HCWs overwhelmingly supported the policy.

**Conclusion:**

Among attendees and HCWs in Rwandan clinics, the acceptability of PITC was very high. PITC appeared to increase testing rates and may be helpful in prevention and early access to treatment.

## Introduction

Knowledge of HIV status may aid in prevention and early treatment. Therefore promotion of Voluntary Counselling and Testing (VCT) is integrated into the national HIV control strategy for many countries with high HIV prevalence. Despite VCT, many people [>61%] in sub-Saharan countries do not know their HIV status [1]–[5]. HIV testing among clinic attendees is low, even in high-risk populations in sub-Saharan Africa [1], [6]–[8]. WHO, UNAIDS and CDC recommend provider-initiated testing and counselling (PITC) in health facilities (HFs) to increase the number of persons who know their HIV status, enable early clinical care and guide provision of specific medical services that would otherwise not be provided [1], [9]. PITC may reduce stigma and discrimination, identifies undiagnosed HIV infection [10] and provides opportunities for women to access HIV testing without seeking permission from their partners [8], [10].

While implementing PITC, patients should be given an option to decline testing; it should not be a medical decision compromising patient’s autonomy [11]–[15]. In many HFs, HIV testing is offered systematically to antenatal care (ANC) attendees and tuberculosis (TB) patients. At out-patient departments (OPD), HIV testing is performed when patients present with symptoms of HIV infection (i.e. for diagnostic purposes). Acceptability of routine HIV testing among ANC [13] and TB patients has been assessed in many studies [16]–[20], however few studies have been conducted among OPD patients [3], [7], [8], [21], [22]. Concerns regarding systematic implementation of PITC such as fear to seek medical care, psychological stress [23], [24], under-resourced providers and facilities remain [25]–[29].

In 2008, the Rwanda Ministry of Health (MOH) adopted PITC as a policy to increase the opportunity for HIV testing and ensure timely HIV diagnosis among HF attendees. Prior to national roll-out of the PITC policy, we implemented a before-and-after controlled intervention study of PITC. We assessed the acceptability of PITC among attendees and health care workers (HCWs), the reasons for being or not being tested for HIV and factors associated with having been tested on the day of the interview among attendees in eight Rwandan HFs.

## Methods

### Setting

Four HFs in Musanze district (North-West Rwanda) and four HFs in Gasabo district (Central Rwanda; area of the capital) were purposefully selected for this study, ensuring inclusion of urban and rural HFs, hospital and clinic settings with sufficient numbers of attendees [Table 1]. HFs had a complete range of HIV testing, care and treatment services. This study consisted of three phases: in phase 1 (March–May 2009) PITC was not operational; in phase 2 (June–November 2009) PITC was introduced; and in phase 3 (December 2009– February 2010) PITC was operational. PITC was introduced in six HFs while two served as controls.

**Table 1:** Characteristics of intervention and control sites, PITC study, Rwanda, 2009–10.

Study site	Province	Level of urbanization	Type of facility	Intervention
Rwaza	Northern	Rural	Health center	Option 1
Kinyinya	Kigali City	Urban	Health center	Option 1
Ruhengeri	Northern	Semi-urban	Hospital	Option 2
Muhoza	Northern	Semi-urban	Health center	Option 2
Kibagabaga	Kigali City	Urban	Hospital	Option 3
Kimironko	Kigali City	Urban	Health center	Option 3
Gasiza	Northern	Rural	Health center	Control
Kabuye	Kigali City	Semi-urban	Health center	Control

Option 1: a rapid test by the HCW using a finger-prick blood sample in the consultation department was done; Option 2: a venous blood sample was drawn by the HCW and sent to the laboratory for rapid testing; Option 3: the HCW offered the test upon consent, and sent the attendee to the laboratory for a venous blood draw and rapid testing.

### Intervention

Biomedical materials for PITC were delivered to six HFs. Health care workers (HCWs) were trained to offer PITC, administer HIV testing, and use registers adapted for the study to record testing. In TB and ANC departments, PITC was already commonly practiced prior to the start of the study, following national policy. During phase 3, PITC was newly operational in the OPD and family planning (FP) departments. HCWs informed clinic attendees that it was MOH-recommended policy to offer HIV testing to all clients. To standardize messaging across intervention sites, HCWs used a local language version of the statement “HIV infection is quite common in Rwanda. Testing for HIV helps people to know their HIV status and thus adopt positive behavior for protecting themselves against acquiring the infection; and for those infected to protect others and start necessary treatment early. In order to give everyone an opportunity to know his/her HIV status we offer systematic HIV testing of all attendees. However, you may choose to decline without any consequences for the care you are entitled to, at this or other health facilities.” This statement was also displayed at HF entrances, in waiting areas and all departments visited by attendees.

PITC was offered in three ways: option 1 involved a rapid test by the HCW using a finger-prick blood sample in the consultation department; option 2 involved a test on a venous blood sample drawn by the HCW and sent to the laboratory for rapid testing; option 3 involved the HCW offering the test, and upon consent sending the attendee to the laboratory for a venous blood draw and rapid testing. In all options, HCWs provided post-test counseling. Each option was implemented in two intervention sites.

We anticipated that this new intervention would increase workload and responsibility for HCWs; accordingly we provided salary top-ups to department heads (150,000 RWF [equivalent to about $225] per month) and stipends to HCWs (50,000 RWF [about $75] per month).

### Selection of Participants and Interviews

Interviews were done in March 2009 and February 2010. Interviewers were trained to systematically select and interview attendees using a schedule, allowing sufficient time for interviews. At exits of HFs, interviewers approached individuals and asked if they would be willing to participate in the study. When an attendee declined participation, the next attendee was approached. Only attendees who had visited HFs seeking their own medical care were eligible for interview. If the attendee was a child (<18 years), the caregiver was interviewed as a proxy. Based on staff lists, a random sample of HCWs for interview was generated using a computer. The selected HCWs were interviewed in phase 1 and 3.

### Questionnaires

The attendee questionnaire addressed: (1) study site, date and time of the interview, and study phase; (2) socio-demographic characteristics of the interviewee (sex, age, education, religion, marital status, occupation), and department attended on the day of interview; (3) outcome variables (knowledge of HIV test and PITC policy, acceptability of PITC and history of HIV testing). PIT policy was explained to attendees: “Since the beginning of 2008, it is recommended by MOH to offer HIV testing to all HF attendees”. Knowledge of an HIV test was assessed by asking: “Do you know what an HIV test is?” Knowledge of PITC policy was assessed by asking: “Had you heard about the PIT policy before?” and acceptability of PITC was assessed by asking: “Do you agree with this recommendation?” Responses were graded on a 5-point scale: “agree strongly”, “agree”, “neutral”, “disagree strongly” and “disagree”. We also asked whether the attendee had been offered an HIV test on the day of interview, had accepted the test, reasons for being or not being tested, and whether they had previously been tested. The interviewer matched the attendees’ responses to pre-coded responses. Among HCWs, we assessed the agreement with PITC policy, and its impact on their workload and duration of patient consultations.

### Data Management and Analysis

Associations between participants’ characteristics and the outcome variables were analysed using chi-squared test, Fisher’s exact test, or rank sum test, as appropriate. From the 5-point scale, the responses “agree strongly” or “agree” were regarded as agreement with the PITC policy. Regarding HIV testing, we distinguished three outcomes: having been tested on the day of the interview; having been tested for HIV before the day of the interview; and ever having been tested (i.e. on day of interview, or before, or both). Independent factors associated with having been tested for HIV on the date of interview were determined using logistic regression models. We restricted the analysis of determinants of HIV testing to attendees of the OPD, as some form of PITC was already practiced in the TB and ANC departments before this study, and the number of attendees in FP departments was rather small. Using the likelihood ratio test, we eliminated variables one by one from the starting model (if p>0.05) until a parsimonious model was obtained. Sex and age group were forced into the models. For HCW interviews, we analyzed the perceptions on PITC acceptability, its impact on routine workload and consultation time. Only HCWs interviewed twice were included in the final analysis. P values of <0.05 were considered statistically significant.

### Ethics

The research ethics committees of Rwanda and the Academic Medical Center, Amsterdam, provided ethical approval. All HF attendees and caregivers [on behalf of children <18 years] are aware that as part of clinical care their demographic and clinical data are registered in paper-based clinic registers. As only such routinely collected data were abstracted from clinic registers, and no names were entered into the electronic study database the ethics committees did not require that written informed consent was sought from patients. All HF attendees and HCWs provided verbal informed consent for participation.

## Results

### Characteristics of the Attendees

We interviewed 1772 clinic attendees: 1365 at intervention HFs (560 in phase 1 and 805 in phase 3) and 407 at control HFs (208 in phase 1 and 199 in phase 3) (Table 2). The median age of attendees visiting intervention HFs was 27 years (IQR 21–35) and the majority (62%) was female. Most were married (56%), farmers by occupation (43%), had at least completed primary school education (70%), and visited the OPD for a clinical consultation (59%) on the day of interview. The phase 1 population differed significantly from the phase 3 population in most respects, but not in age (p = 0.34) and sex (p = 0.06). Of the 407 attendees interviewed at the two control HFs, the median age was 28 years (IQR 22–36) and the majority (58%) was female. Attendees did not differ with respect to sex (p = 0.19) but differed in age (p = 0.047) between intervention and control HFs.

**Table 2:** Characteristics of clinic attendees interviewed at intervention and control sites, PITC study, Rwanda, 2009–10.

Characteristic	Intervention population			Control population		
	Phase 1	Phase 3		Phase 1	Phase 3	
	N = 560	N = 805	P	N = 208	N = 199	P
**Sex**			0.06			0.17
Male	197 (35.2%)	323 (40.2%)		80 (38.5%)	90 (45.2%)	
Female	363 (64.8%)	480 (59.8%)		128 (61.5%)	109 (54.8%)	
Missing	–	2				
**Median age (IQR) in years**	27 (22–36)	27 (20–34)	0.34	29 (22–38)	28 (22–36)	0.41
**Age-group (in years)**			0.15			0.81
0–4	39 (7.0%)	50 (6.3%)		12 (5.8%)	18 (9.1%)	
5–14	25 (4.5%)	61 (7.7%)		11 (5.3%)	10 (5.1%)	
15–24	150 (26.8%)	200 (25.2%)		50 (24.0%)	42 (21.2%)	
25–34	195 (34.8%)	288 (36.3%)		71 (34.1%)	74 (37.4%)	
35–44	84 (15.0%)	104 (13.1%)		33 (15.9%)	25 (16.6%)	
45–54	44 (7.9%)	48 (6.1%)		15 (7.2%)	13 (6.6%)	
≥55	23 (4.1%)	42 (5.3%)		16 (7.7%)	16 (8.1%)	
Missing	–	12		–	1	
**Marital status**			0.001			0.18
Single	181 (32.6%)	297 (37.3%)		53 (25.7%)	68 (34.2%)	
Married	308 (55.5%)	451 (56.7%)		137 (66.5%)	117 (58.8%)	
Divorced/Widowed	66 (11.9%)	48 (6.1%)		16 (7.8%)	14 (7.0%)	
Missing	5	9		2	–	
**Occupation**			0.013			0.51
Farmer	225 (43.6%)	312 (43.0%)		125 (66.5%)	105 (60.0%)	
Laborer	70 (13.6%)	66 (9.1%)		26 (13.8%)	26 (14.9%)	
Other	174 (33.7%)	249 (34.3%)		28 (14.9%)	36 (20.6%)	
Student	47 (9.1%)	99 (13.6%)		9 (4.8%)	8 (4.6%)	
Missing	44	79		20	24	
**Religion**			<0.001			0.001
Catholic	255 (45.6%)	465 (57.9%)		105 (50.5%)	125 (62.8%)	
Other Christian	264 (47.2%)	283 (35.2%)		68 (32.7%)	62 (31.2%)	
Other	40 (7.2%)	55 (6.9%)		35 (16.8%)	12 (6.0%)	
Missing	1	2				
**Education**			<0.001			<0.001
None	204 (36.5%)	182 (18.1%)		121 (58.5%)	54 (27.1%)	
Primary	285 (51.0%)	428 (53.8%)		77 (37.2%)	104 (52.3%)	
Secondary	70 (12.5%)	169 (21.3%)		9 (4.4%)	41 (20.6%)	
Missing	1	10		1	–	
**Study site**			<0.001			0.005
Ruhengeri	75 (13.4%)	110 (13.7%)		–	–	
Muhoza	75 (13.4%)	149 (18.5%)		–	–	
Kibagabaga	101 (18.0%)	146 (18.1%)		–	–	
Kinyinya	119 (21.3%)	100 (12.4%)		–	–	
Kimironko	115 (20.5%)	150 (18.6%)		–	–	
Rwaza z	75 (13.4%)	150 (18.6%)		–	–	
Gasiza	–	–		75 (36.1%)	99 (49.8%)	
Kabuye	–	–		133 (63.9%)	100 (50.3%)	
**Department visited**			<0.001			<0.001
OPD	289 (51.6%)	521 (64.7%)		107 (51.4%)	153 (76.9%)	
FP	70 (12.5%)	107 (13.3%)		25 (12.0%)	17 (8.5%)	
TB	14 (2.5%)	23 (2.9%)		2 (1.0%)	–	
VCT	60 (10.7%)	48 (6.0%)		39 (18.8%)	18 (9.1%)	
ANC	57 (10.2%)	72 (8.9%)		35 (16.8%)	11 (5.5%)	
Other	70 (12.5%)	34 (4.2%)		–	–	

PITC: provider initiated testing and counselling; IQR: Inter-quartile range; OPD: out-patient department; FP: family planning; VCT: Voluntary counseling and testing; ANC: Antenatal care; TB: tuberculosis.

P values are based on the chi-squared test, except comparison of age (based on rank sum test).

Phase 1: before PITC was implemented; Phase 3: after PITC was implemented (at the intervention sites).

### PITC Acceptability

At intervention sites, 93% of attendees during phase 1 knew what an HIV test was and this increased to 98% during phase 3 (p<0.001; Table 3). The proportion of attendees that had heard about PITC policy increased from 19% to 68% (p<0.001). The acceptability of PITC was 97% during phase 1, and 99% during phase 3 (p = 0.018). At control sites, 72% knew what an HIV test was in phase 1, which increased to 81% in phase 3 (p = 0.028). Twenty-six percent and 33% had knowledge about PITC in phase 1 and 3 respectively (p = 0.17). The acceptability of PITC was 97% in phase 1 and 98% phase 3 (p = 0.57).

**Table 3:** Outcomes from interviews of clinic attendees at intervention and control sites, PITC study, Rwanda, 2009–10.

Interview outcomes	6 intervention sites			2 Control sites		
	Phase 1	Phase 3	P	Phase 1	Phase 3	P
	N = 560 (%)	N = 805 (%)		N = 208 (%)	N = 199 (%)	
**Does the attendee know what an HIV test is?**			<0.001			0.028
Yes	520 (92.9%)	789 (98.0%)		149 (71.6%)	161 (80.9%)	
No	40 (7.1%)	16 (2.0%)		59 (28.4%)	38 (19.1%)	
**Did the attendee hear about the Rwandan PIT policy before?**			<0.001			0.17
Yes	108 (19.3%)	536 (68.0%)		55 (26.4%)	65 (32.7%)	
No	452 (80.7%)	252 (32.0%)		153 (73.6%)	134 (67.3%)	
**Does the attendee agree with the PIT policy?**			0.018			0.57
Agree*	544 (97.3%)	794 (99.0%)		202 (97.1%)	195 (98.0%)	
Disagree	15 (2.7%)	8 (1.0%)		6 (2.9%)	4 (2.0%)	

PITC: provider initiated testing and counselling; Phase 1: before PITC; Phase 3: PITC phase;

*the results reflect the perceptions from the interviews.

### PITC’s Impact on HIV Testing Rates

The proportion of attendees who reported to have been tested on the day of interview during phase 3 (33%) was significantly higher than during phase 1 (24%) in intervention sites (p<0.001, Table 4). The proportion tested before the day of interview was 77% in phase 1 and 85% in phase 3 (p<0.001). The proportion of attendees reporting ever having been tested was 82% in phase 1 and 92% in phase 3 (p<0.001). Among attendees of control HFs, the proportions of attendees tested on the day of interview were 30% in phase 1 and 18% in phase 3 (p = 0.003). Attendees who reported having been tested before were 70% and 72% (p<0.71); and the proportions of attendees having ever been tested (including on day of interview) were 77% and 74% (p = 0.48).

**Table 4:** Proportions of health care facility attendees who were tested on day of interview, tested for HIV before and had ever been tested”, by clinic department for the intervention and control sites, PITC study, Rwanda, 2009–10.

	Tested for HIV on day of interview			Tested for HIV before			Ever Tested for HIV		
Department	Phase 1	Phase 3	P	Phase 1	Phase 3	P	Phase 1	Phase 3	P
Intervention	N = 560	N = 805		N = 560	N = 805		N = 560	N = 805	
**All**	134/560 (23.9%)	268/805 (33.3%)	<0.001	432/560 (77.1%)	687/805 (85.3%)	<0.001	460/560 (82.1%)	738/805 (91.7%)	<0.001
OPD	34/289 (11.8%)	117/521 (22.5%)		204/289 (70.6%)	421/521 (80.8%)		213/289 (73.7%)	457/521 (87.7%)	
FP	6/70 (8.6%)	30/107 (28.0%)		69/70 (98.6%)	105/107 (98.1%)		69/70 (98.6%)	107/107 (100%)	
TB	1/14 (7.1%)	–		14/14 (100%)	22/23 (95.7%)		14/14 (100%)	22/23 (95.7%)	
VCT	57/60 (95.0%)	48/48 (100%)		47/60 (78.3%)	38/48 (79.2%)		60/60 (100%)	48/48 (100%)	
ANC	32/57 (56.1%)	62/72 (86.1%)		52/57 (91.2%)	70/72 (97.2%)		57/57 (100%)	72/72 (100%)	
Other	4/70 (5.7%)	11/34 (32.4%)		46/70 (65.7%)	31/34 (91.2%)		47/70 (67.1%)	32/34 (94.1%)	
**Control**	**N = 208**	**N = 199**		**N = 208**	**N = 199**		**N = 208**	**N = 199**	
**All**	63/208 (30.3%)	35/199 (17.6%)	0.003	146/208 (70.2%)	143/199 (71.9%)	0.71	161/208 (77.4%)	148/199 (74.4%)	0.48
OPD	9/107 (8.4%)	8/153 (5.2%)		57/107 (53.3%)	101/153 (66.0%)		61/107 (57.0%)	102/153 (66.7%)	
FP	2/25 (8.0%)	2/17 (11.8%)		24/25 (96.0%)	17/17 (100%)		24/25 (96.0%)	17/17 (100%)	
TB	–	–		2/2 (100%)	–		2/2 (100%)	–	
VCT	38/39 (97.4%)	18/18 (100%)		31/39 (79.5%)	16/18 (88.9%)		39/39 (100%)	18/18 (100%)	
ANC	14/35 (40.0%)	7/11 (63.6%)		32/35 (91.4%)	9/11 (81.8%)		35/35 (100%)	11/11 (100%)	

Intervention: intervention sites; Control: control sites; PITC: provider initiated testing and counselling; Phase 1: before PITC; Phase 3: PITC phase; OPD: outpatient department; FP: family planning; VCT: voluntary counseling and testing; ANC: antenatal care; TB: tuberculosis. Note: for this table we assumed that the patients of whom information on HIV testing on date of interview (n = 61; 56 from OPD and 5 from FP) or ever before (n = 4; all from OPD) was lacking, had not been tested. “Ever tested for HIV” means being tested for HIV on day of interview, or having been tested for HIV before, or both.

### Reasons for Being Tested or Not

The reasons for being tested for HIV and reasons for not being tested differed between and phases 1 and 3 at the intervention sites. In phase 1, the most frequent reason for testing was the desire to know one’s HIV status (64%) while during phase 3, the most frequent reason was an HIV test offer by an HCW (69%) (Table 5). Among attendees at control sites a similar shift in reasons for being tested was observed. At intervention sites, the most frequent reason for not having been tested was prior knowledge of one’s HIV status (whether positive or negative); this constituted 39% during phase1 and 58% during phase 3). The second most frequent reason was lack of an HIV test offer by the HCW (25% in phase 1 and 18% in phase 3). At control sites, prior knowledge and lack of HIV test offer by the HCW were the two most frequent reasons for not having an HIV test in both phase 1 and phase 3.

**Table 5:** Reasons for having been tested or not having been tested for HIV among health facility attendees by study phase, PITC study, Rwanda, 2009–10.

	Intervention sites		Control sites	
	Phase 1	Phase 3	Phase 1	Phase 3
Reasons for having been tested for HIV	N = 134 n(%)	N = 268 n (%)	N = 63 n (%)	N = 35 n (%)
Personal knowledge/curiosity	82 (64.1%)	60 (23.9%)	45 (77.6%)	15 (42.9%)
Offer by HCW	19 (14.8%)	172 (68.5%)	10 (17.2%)	20 (57.1%)
Administrative reason	14 (10.9%)	16 (6.4%)	–	–
Marriage	7 (5.5%)	1 (0.4%)	1 (1.7%)	–
Positive family member/suspect	2 (1.7%)	1(0.4%)	–	–
Tb infection/suspect	1 (0.8%)	1 (0.4%)	1 (1.7%)	–
Unprotected sex	1 (0.8%)	–	1 (1.7%)	–
Post-exposure	1 (0.8%)	–	–	–
Positive family member	1 (0.8%)	–	–	–
**Reasons for not having been tested for HIV**	**N = 420 n (%)**	**N = 482 n (%)**	**N = 144 n (%)**	**N = 158 n (%)**
Knowing status already	157 (38.6%)	277 (58.4%)	50 (34.7%)	66 (41.8%)
Never offered by HCW	100 (24.6%)	83 (17.5%)	40 (27.8%)	49 (31.0%)
Other	72 (17.7%)	31 (6.5%)	23 (16.0%)	13 (8.2%)
Don't know reason	36 (8.9%)	20 (4.2%)	3 (2.1%)	1 (0.6%)
Does not feel at risk	29 (7.1%)	37 (7.8%)	21 (14.6%)	22 (13.9%)
Not interested/willing	10 (2.5%)	7 (1.5%)	4 (2.8%)	1 (0.6%)
Too weak	2 (0.5%)	10 (2.1%)	–	–
Fear of discrimination	1 (0.3%)	1 (0.2%)	–	2 (1.3%)
Afraid of test result/HIV status	–	1 (0.2%)	1 (0.7%)	2 (1.3%)
Too tired	–	7 (1.5%)	–	–
Fear of pain	–	–	2 (1.4%)	2 (1.3%)

PITC: provider initiated testing and counseling; HCW: health care worker; Phase 1: before provider initiated testing; Phase 3: Intervention phase.

### Factors Associated with HIV Testing on the Day of Interview in OPD

In bivariable analysis PITC, age, religion and education were significantly associated with testing on the day of interview among OPD patients (Table 6). In multivariable analysis, factors significantly associated with testing on the day of interview were PITC (OR = 2.8, 95% CI = 1.8–4.2 during phase 3 compared to phase 1), age (≥15 years compared to those below) and not having been previously tested (OR = 2.1, 95% CI = 1.4–3.3).

**Table 6:** Association between having been tested for HIV on day of interview and demographic characteristics and testing history among 1009 attendees of the OPD at 8 clinics, Rwanda, 2009–10.

Characteristic	n/N (%)	OR (95%CI)	P	aOR (95%CI)	P
**PITC**			<0.001		<0.001
PITC sites phase 1	34/283 (12.0%)	1		1	
PITC sites phase 3	117/471 (24.8%)	2.4 (1.6–3.7)		2.8 (1.8–4.2)	
Control sites phase 1	9/106 (8.5%)	0.7 (0.3–1.5)		0.6 (0.3–1.4)	
Control sites phase 3	8/149 (5.4%)	0.4 (0.2–0.9)		0.4 (0.2–0.9)	
**Sex:**			0.27		0.22
Male	76/496 (15.3%)	1		1	
Female	92/513 (17.9%)	1.2 (0.9–1.7)		1.3 (0.9–1.8)	
**Age-group (in years)**			0.006		<0.001
0–4	9/106 (8.5%)	1		1	
5–14	7/88 (8.0%)	0.9 (0.3–2.6)		0.8 (0.3–2.3)	
15–24	51/220 (23.2%)	3.3 (1.5–6.9)		4.3 (1.9–9.6)	
25–34	50/301 (16.6%)	2.1 (1.0–4.5)		2.9 (1.3–6.5)	
35–44	23/125 (18.4%)	2.4 (1.1–5.5)		3.4 (1.4–8.3)	
45–54	14/89 (15.7%)	2.0 (0.8–4.9)		3.1 (1.2–8.0)	
≥55	13/76 (17.1%)	2.2 (0.9–5.5)		2.8 (1.1–7.2)	
**Marital status**			0.55		
Single	67/439 (15.3%)	1			
Married	84/477 (17.6%)	1.2 (0.8–1.7)			
Divorced/Widowed	16/85 (18.8%)	1.3 (0.7–2.4)			
**Occupation**			0.09		
Cultivator	81/405 (20.0%)	1			
Laborer	16/112 (14.3%)	0.7 (0.4–1.2)			
Other	33/228 (14.5%)	0.7 (0.4–1.1)			
Student	28/118 (23.7%)	1.2 (0.8–2.0)			
**Religion**			0.027		
Catholic	113/587 (19.3%)	1			
Other Christian	42/335 (12.5%)	0.6 (0.4–0.9)			
Other	13/84 (15.5%)	0.8 (0.4–1.4)			
**Education**			0.020		
None	45/359 (12.5%)	1			
Primary	90/460 (19.6%)	1.7 (1.2–2.5)			
Secondary	33/178 (18.5%)	1.6 (1.0–2.6)			
**Tested before date of interview**			0.57		0.001
Yes	118/727 (16.2%)	1		1	
No	50/278 (18%)	1.1 (0.8–1.6)		2.1 (1.3–3.3)	

PITC: provider initiated testing and counselling; at control site there was no PIT; OR Odds ratio; aOR: adjusted odds ratio; OPD: out-patient department; P values are based on chi-squared tests of univariable logistic regression analysis and likelihood ratio tests for multivariable logistic regression analysis.

### Results from HCWs Interviews

Eighty-five HCWs were interviewed twice, 72 from intervention and 13 from control HFs. The median age was 33 years (IQR 30–38) and the majority 74% (63/85) were female. Most were nurses (71%; 60/85). The acceptability of the PITC policy was nearly universal: only one HCW from a control site (phase 3) disagreed. At the intervention sites, 67% (48/72) of HCWs reported working for >40 hours per week in the seven days preceding the phase 1 interview, this increased to 85% (61/72) during phase 3. Similarly, the average duration of consultation time >15 minutes was reported by 26% (19/72) of the HCWs during phase 1, which increased to 43% (31/72) during phase 3. There were no changes in either workload or consultation time reported at control sites.

## Discussion

### Main Findings

PITC is acceptable to the majority of attendees and HCWs in Rwandan HFs. Desire to know one’s HIV status and having been offered an HIV test by a HCW were the most frequent reasons for testing. Routine PITC appeared to increase testing uptake at the OPD. Not having been previously tested and age above 15 years were other factors significantly associated with testing on the day of interview.

### PITC Acceptability

The acceptability of PITC was high among clients who attended HFs implementing PITC, as well as among clients of HFs without PITC. This finding suggests that the intervention did not lead to a reduction in acceptability. Although there is limited literature on patient perceptions of PITC [25], our data concur with the results from the few studies in which PITC acceptability was assessed. In Botswana 81% of participants reported being extremely in favor of routine HIV testing [9] while 94% of household respondents reported being in favor of routine HIV testing in government HFs [30]. A study from Zambia reported 96% of patients being in favor of PITC [8].

### Reasons for Being Tested or Not

Where PITC was implemented, the offer of the test by a HCW was the most common reason for testing on the day of interview. Steen et al reported that the main reasons for accepting routine HIV testing were patients’ wish, pregnancy, medical examination, clinical suspicion and sexually transmitted diseases [31]. In our study, prior knowledge of HIV status was the most common reason for not being tested, in line with studies from Uganda and South Africa [32], [33]. In Rwanda the MOH’s advice is that an HIV test may be repeated when three months have passed since the last negative HIV test. Unexpectedly, 11% of attendees of HFs where PITC was implemented reported they had not been offered an HIV test. Another finding is that despite the high acceptability of PITC, the actual testing rate on the day of interview was low. This may be explained by the increase in the proportion of clinic attendees that reported previous HIV testing.

### Impact of PITC on HIV Testing Rates

At intervention sites a higher proportion of attendees was tested on the day of interview during PITC roll-out compared to phase 1 (33% vs. 24%). By contrast, attendees at control sites were less likely to have been tested on the day of interview during phase 3 compared phase 1 (18% vs. 30%). This apparent decline at the control sites is explained by differences in composition of the attendee population at the control sites between phases 1 and 3 (relatively larger proportion of OPD attendees in phase 3 than in phase 1); testing rates did not decline significantly in any of the departments at control sites. The Rwanda Demographic and Health Survey 2010 reported 76% of women and 69% of men had ever been tested and received their test results [34] while 92% of attendees reported ever having been tested from our study. Our findings suggest increased HIV testing uptake among HF attendees due to routine PITC. Other studies from sub-Saharan Africa reported similar results [7], [8], [23].

### Factors Associated with HIV Testing on the Day of Interview in OPD

Attendees who visited the OPD in phase 3 had more than two-fold increased odds of being tested for HIV compared to those who visited an OPD with PITC in phase 1. The odds for having been tested for attendees at control sites were similar in phases 1 and 3. Those who had not been tested before were more likely to be tested on the day of interview. In a study from Botswana, female sex, education, multiple visits to a medical doctor, and perceived access to good quality services, were significant predictors of accepting an HIV test [10]. In our study, neither sex nor education was significantly associated with testing. A review indicated that provider factors (constraints of training, time and resources), client factors (cost and transport obstacles) and the level of trust in provider-client relationship all affected the utilization of HIV testing services at HFs [6].

### PITC’s Impact on HCWs’ Workload

The general perception among HCWs was that routine PITC increased their workload. Our results indicate an increase in both total weekly working time (proportion >40 hours) and consultation time per attendee (proportion >15 minutes). Based on these changes, and the reported two-fold likelihood of being tested at intervention HFs, it appears that PITC increased both staff workload and the waiting time for patients. Studies in sub-Saharan Africa reported increased workload and occupational stress [27] and patient waiting time [26]. A study on PITC from Zambia also reported a three-fold increase in the number of patients tested per HCW due to opt-out-testing compared to standard non-PITC [8]. For these reasons, Roura et al [29] report the importance for additional resources and efforts to effectively implement routine PITC.

### Limitations

Study sites were not chosen randomly and may not be representative. Testing history was based on self-report and may have been affected by recall bias or social desirability bias. Phase 3, during which PITC was implemented was a different season than phase 1; this may have affected the composition of the group of attendees, and hence attitudes and testing history. The increase in knowledge on HIV testing from phase 1 to phase 3 may be a seasonal or secular effect, as also the knowledge among attendees of control sites increased. As these effects would have similarly affected control sites, and we controlled for several key demographic variables in the multivariable analysis of determinants for HIV testing on day of interview, we think this possible seasonal effect was controlled for.

### Conclusion

Among attendees and HCWs in Rwandan HFs, the acceptability of PITC was very high. Having been offered an HIV test and the attendee’s desire to know their HIV status were the most common reasons for testing. PITC is acceptable and appeared to increase HIV testing uptake, but increased workload. PITC may be helpful in prevention and increasing early access to treatment.
